# Associations Between Relative Age, Siblings, and Motor Competence in Children and Adolescents

**DOI:** 10.3390/children12050563

**Published:** 2025-04-26

**Authors:** Daniel González-Devesa, José Carlos Diz-Gómez, Pedro Vicente-Vila, Marta Domínguez Fernández, Miguel Rodríguez Rodríguez, Rocío Carballo-Afonso, Miguel Adriano Sanchez-Lastra, Carlos Ayán-Pérez

**Affiliations:** 1 Grupo de Investigación en Actividad Física, Educación, y Salud (GIAFES), Universidad Católica de Ávila, C/Canteros, 05005 Ávila, Spain; 2 Well-Move Research Group, Galicia Sur Health Research Institute (IIS Galicia Sur), SERGAS-UVIGO, 36310 Vigo, Spain; jcdiz@uvigo.es (J.C.D.-G.); misanchez@uvigo.gal (M.A.S.-L.); cayan@uvigo.es (C.A.-P.); 3 Departamento de Didácticas Especiáis, Universidad de Vigo, 36310 Vigo, Spain; pachipedrito@edu.xunta.gal (P.V.-V.); rociocarballo@uvigo.gal (R.C.-A.); 4 Facultad de Ciencias de la Educación y del Deporte, Universidad de Vigo, 36005 Pontevedra, Spain; martadominguezfernandez01@gmail.com (M.D.F.); miguii.rodriguez.20@gmail.com (M.R.R.)

**Keywords:** youth, exercise, physical development, physical fitness, exercise test

## Abstract

Background: Motor competence is a key determinant of children’s physical, cognitive, and social development, and it is influenced by various biological and environmental factors. Among these, relative age and the presence of siblings have been proposed as potential contributors, yet their impact remains underexplored, especially in school-aged populations. Objective: This study investigates the influence of relative age and the effects of the presence of siblings on the motor competence of children and adolescents. Methods: The motor competence level of 747 students, 381 from primary school (mean age = 8.81 ± 1.8 years) and 366 from secondary school (mean age = 13.52 ± 1.22 years), was measured by the means of the Canadian Agility and Movement Skill Assessment. Participants were queried about their birth date and whether they had any older siblings. Results: Overall, motor competence exhibited an upward trend with chronological age, reaching its peak among children aged 14 years old. Analysis revealed a significant impact of relative age effects on motor competence among primary children, with considerably higher scores in the first semester (*p* = 0.017). Conversely, no clear trend was observed for secondary children. Having siblings did not significantly affect motor competence proficiency. Multiple regression analysis further confirmed that relative age did not contribute to this lack of association (quarter of birth: *p* = 0.003; β = −0.144; siblings: *p* = 0.697; β = −0.019). Conclusions: These findings suggest that physical education teachers should be aware of the influence of relative age not only when assessing motor competence but also when designing and implementing their teaching practice in primary school settings. In contrast, the effect of relative age appears to be less significant in secondary education, where it may be less relevant for both evaluation and teaching practice. Further research should explore longitudinal designs and consider additional psychosocial and environmental variables to better understand how these factors influence motor competence development over time.

## 1. Introduction

Motor competence (MC) is defined as the level of proficiency with which children execute fundamental motor skills (FMSs) [1]. Both FMSs and the resulting MC have been consistently linked to favourable health outcomes, including higher levels of physical activity and fitness, greater perceived competence, improved cognitive performance, and healthier body mass [2,3]. Additionally, the level of MC is a key factor in children’s psychosocial attributes, as evidence suggests that low MC can adversely affect their social interactions and emotional well-being [4].

Given the relevance of MC, a considerable amount of research has focused on understanding its developmental trajectory and identifying the factors that influence it. The development of MC is influenced by both internal and external factors. Internal, or genetic, components determine an individual’s natural potential, while external factors are shaped by the surrounding environment [5,6]. External influences can include where a child lives, the family’s involvement in sports, economic background, and parents’ education levels. Within this framework, particular attention has been paid to biological and demographic variables [7], among which relative age and the presence of siblings have emerged as notable areas for further investigation.

Relative age refers to the age disparities among individuals grouped within the same annual cohort [8]. It is commonly defined as the age difference between individuals born within a designated cut-off period [9]. The phenomenon known as the relative age effect (RAE) has been extensively examined in the context of sports [10], with findings consistently indicating a competitive advantage for those born earlier in the selection year [11]. Several studies have also confirmed the presence of the RAE in relation to physical fitness in children and adolescents [12,13,14].

However, research exploring the influence of the RAE on MC has been predominantly focused on preschool-aged children, with limited evidence available for students in primary and secondary education. While most existing studies have investigated this relationship in primary school populations [15,16,17], research involving secondary school students remains scarce. This is a matter of concern, since the presence of the RAE in physical education has been confirmed not only for primary but also for secondary students [18,19]. Previous research has indicated that primary school physical education teachers should be aware of the RAE on motor skills for evaluation purposes [15], but it is not known whether the same applies for secondary students. Finally, it should be highlighted that none of these studies on the relationship between RAE and MC have taken into account the presence of siblings as a confounding variable, despite being recognized as an important developmental agent [20].

The presence of siblings has been proposed as a potential factor influencing the development of MC [21]. Notably, family dynamics—specifically the number of siblings and one’s position in the birth order—play a significant role [22]. Siblings often contribute to each other’s growth through shared experiences and interactions, though their impact can be either supportive, by acting as positive role models, or detrimental, when rivalry for attention and resources arises [23]. Older siblings often serve as more advanced developmental models for their younger counterparts, contributing to the creation of a stimulating and enriched environment that may facilitate the younger children’s motor development [23]. Empirical evidence has demonstrated that, during childhood, having siblings is positively associated with increased levels of physical activity and physical fitness [24]. In this sense, McHale et al. [25] emphasized that having an older sibling can offer younger children valuable learning opportunities through imitation and observation, potentially enhancing their development. In this context, it has been noted that the role of older siblings in promoting development, offering new experiences, and altering the environment for younger siblings is an interesting area of research. This suggests that older siblings act as role models for introducing new motor activities [26]. However, limited research has explored the specific effect of siblings on MC [27], and the available findings, primarily derived from studies involving infants and preschool-aged children, have produced inconsistent results [28]. Consequently, there is a lack of conclusive evidence regarding the influence of siblings on the motor competence of children in primary and secondary education.

In light of these considerations, the present study seeks to examine the effect of the RAE on the motor competence levels of primary and secondary school students, while also accounting for the presence of siblings as a relevant developmental variable. In the context of the existing literature, it is hypothesized that the RAE and having older siblings have a positive impact on MC.

## 2. Materials and Methods

### 2.1. Participants

The present study involved a total of 747 students, comprising 381 primary school children (mean age = 8.81 ± 1.8 years) and 366 secondary school students (mean age = 13.52 ± 1.22 years). Participants were healthy children aged between 6 and 16 years, recruited from six schools located in urban areas of northern Spain. A convenience sampling strategy was employed, based on the voluntary participation and availability of the selected schools. Eligibility criteria required participants to be free from any medical condition that might have hindered their ability to perform the field-based assessments. Children with physical or intellectual disabilities that could have compromised their comprehension of the testing protocols or interfere with their ability to execute the required tasks were excluded from this study. Prior to participation, written informed consent was obtained from the parents or legal guardians of all students. The research protocol received ethical approval from the Ethics Committee of the Faculty of Education and Sport Sciences at the University of Vigo (Code: 03-170123; Date of Approval: 17 January 2023).

### 2.2. Measurements

#### 2.2.1. Relative Age and Siblings

Information regarding participants’ dates of birth and the presence of older siblings was collected through direct questioning. These data were obtained by the physical education teachers responsible for administering the assessments.

#### 2.2.2. Anthropometry

Body height was measured using a Stanley PowerLock (Stanley Black & Decker, New Britain, CT, USA) tape measure, and body mass was assessed with a Body Composition Monitor BF511 (Omron, Kyoto, Japan). Body mass (kg) and body height (cm) were measured without shoes and with light clothing. Each child’s body mass index (BMI) was calculated using the following formula: body mass/body height^2^ (kg/m^2^).

#### 2.2.3. Motor Competence

The Canadian Agility and Movement Skill Assessment (CAMSA) was used to assess MC [29]. This circuit-based assessment integrates both product- and process-oriented metrics, allowing for a comprehensive analysis of fundamental, complex, and combined motor skills. Its application in Spanish children and adolescents has demonstrated high levels of feasibility, validity, and reliability [30].

The CAMSA measures children’s execution of seven distinct motor skills, emphasizing both speed and precision. The tasks include two-foot jumping (2 points), sliding (3 points), catching (1 point), throwing (2 points), skipping (2 points), one-foot hopping (2 points), and kicking (2 points). The process-oriented evaluation yields a skill score derived from the number of correctly performed movements, with a possible range from 0 to 14 points. In addition, the time taken to complete the circuit is recorded and converted into a time score ranging from 1 to 14 points. The total score (CAMSA-S) is the sum of both components, producing a final result between 1 and 28 points, where higher scores indicate better motor competence [31].

Following the standardized procedure, participants first completed two practice trials, followed by two official trials that were timed and scored. The assessment was conducted under the supervision of two trained examiners. The first examiner was tasked with providing the ball, delivering verbal instructions, and recording completion time, while the second examiner was responsible for evaluating the quality of the movements performed. Both examiners had received prior training in the administration of the CAMSA and followed the official CAPL-2 manual.

### 2.3. Statistical Analysis

The statistical analysis was performed using the SPSS software, version 24 (Armonk, NY, USA: IBM Corp.). The normality of the data distribution was evaluated using the Kolmogorov–Smirnov test. Quantitative variables were expressed as the mean and standard deviation or as median and interquartile range (IQR), depending on the distribution, while qualitative variables were reported as absolute frequencies and percentages.

Given that CAMSA scores did not follow a normal distribution, non-parametric tests were applied for analyses involving these variables. In order to account for the known effects of age and sex on CAMSA performance, percentile scores were calculated and stratified accordingly, allowing for comparisons across birth trimesters. The Kruskal–Wallis test was used to compare CAMSA percentiles among the different trimesters of birth, while the Mann–Whitney U test was applied to compare scores between birth semesters. To examine associations between variables, Spearman’s rank correlation coefficient was employed. Furthermore, a multiple linear regression analysis was conducted to explore the interaction between birth trimester and the presence of siblings, using the CAMSA score as the dependent variable.

## 3. Results

A total of 747 students participated in this study, comprising 381 primary school pupils (mean age: 8.81 ± 1.8 years; 54.1% boys) and 366 secondary school pupils (mean age: 13.52 ± 1.22 years; 49.7% boys), all of whom completed the CAMSA. Table 1 presents the CAMSA scores disaggregated by age and educational stage. In general, performance on the CAMSA demonstrated a progressive increase with chronological age, peaking at 14 years. Additionally, a statistically significant inverse correlation was observed between the body mass index and CAMSA score (Spearman’s Rho = −0.185; *p* = 0.001).

Table 2 displays the data related to relative age, stratified by educational level. The analysis indicated a statistically significant effect of the RAE on CAMSA performance among primary school children, with higher scores observed in those born during the first semester of the year (*p* = 0.017). In contrast, no consistent pattern was identified among secondary school students. Spearman’s correlation supported the presence of a relative age effect in the primary education group, although the association was of a low magnitude (rho = −0.117; *p* = 0.030). In the case of secondary students, no evidence of a relative age effect was found (rho = 0.22; *p* = 0.572).

**Figure 1:**
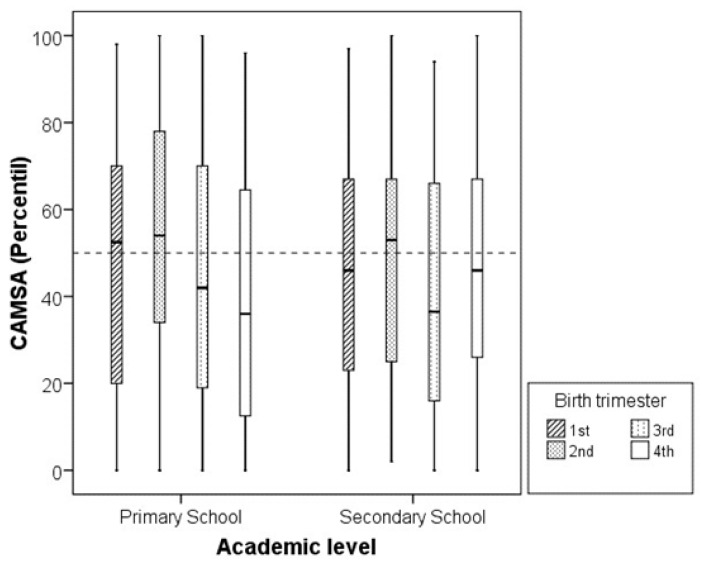
Box plot of CAMSA score percentile (normalized by age and sex) according to birth trimester and educational level. Boxes represent median and interquartile range; whiskers represent extreme values. Dashed line indicates median value (50th percentile).

The distribution of CAMSA scores by birth quarter is illustrated in Figure 1. Overall, birth quarter had only a modest impact on CAMSA performance when normalized by age and sex. Median CAMSA percentiles for all birth trimesters clustered near the 50th percentile, with first- and second-trimester births showing slightly higher medians than those born later in the year at both the primary and secondary school levels. The interquartile ranges demonstrated similar variability in children’s motor competence across both school levels.

Sibling data were collected from a subset of 432 participants (312 primary and 120 secondary students), since not all PE teachers participating in the research were able to collect and complete all the intended data, among whom 34.5% acknowledged having older sisters or brothers. A reduced percentage of primary school children acknowledged having siblings. The comparative analyses revealed that the presence of siblings did not have a statistically significant effect on CAMSA performance, either in the overall sample or when stratified by educational stage and sex. Furthermore, the multiple regression analysis confirmed that relative age did not explain the absence of this association. While the quarter of birth showed a significant effect (*p* = 0.003; β = −0.144), the presence of siblings did not demonstrate a statistically significant contribution to CAMSA scores (*p* = 0.697; β = −0.019).

## 4. Discussion

The aim of this study was to examine the influence of the RAE on motor competence levels among primary and secondary school students, while also accounting for the potential role of having older siblings. The results obtained may offer meaningful implications for physical education teachers in both educational stages, particularly regarding the need to consider alternative assessment strategies that go beyond traditional, norm-referenced approaches. Furthermore, acknowledging the impact of relative age can assist physical education professionals in refining their evaluation criteria and in designing inclusive, developmentally appropriate activities that foster motor competence in all students, regardless of their birth month. This study also contributes to the growing body of the literature focused on the correlates and determinants of motor competence—a research area that continues to evolve and expand [32].

The presence of the RAE in the motor competence levels of primary school children was observed. However, the associations between relative age and CAMSA scores in the participants were weak. Previous research with similar aims has reported inconsistent findings. Birch et al. [17] conducted an assessment involving 539 children aged 6 to 11 years who performed an FMS battery. They reported that children born in the first quarter of the school year exhibited better mastery of object control, and significant differences were found favouring boys. However, no other RAE was identified for FMSs like balance or galloping. In this line, Jarvis et al. [15], after assessing the FMSs of 560 children aged 10–11 years using a comprehensive battery, observed that the RAE occurred solely in boys and only for skills requiring object control. Mixed results were also documented by McPhillips and Jordan-Black [33] in a study involving 1124 elementary and primary school children who underwent the “catch the ball” test. The results indicated a significant effect of the month of birth for Year 3 pupils but not for Year 5 and 7 pupils. Finally, in a study similar to the present one, Dutil et al. [16] administered the CAMSA with 8–12-year-old children (n = 8044) and observed the existence of the RAE. Nevertheless, significant associations were noted to have only negligible effect sizes. In the research, as in the present study, no gender differences were observed. Altogether, these findings imply that the RAE exists in primary children, but it seems to be of a low magnitude, at least when it comes to assessing MC by a single test.

This appears to be the first study to report empirical evidence on the relationship between the RAE and motor competence in secondary school students. Although motor competence undergoes a critical developmental phase during early childhood, its progression continues throughout middle and late childhood, extending up to approximately 18 years of age [34]. Thus, it is relevant to investigate whether the influence of the RAE persists during adolescence. The findings of this study indicate that such an influence is not evident at the secondary education level, supporting the view that RAEs are more prominent during the earlier years of schooling [35]. Notably, previous research examining the association between relative age and physical fitness has shown that the RAE remains observable up to the ages of 12 to 14 but tends to decline thereafter [36] and may even disappear entirely [12]. Collectively, these results suggest that the RAE on motor competence diminishes following the onset of physical maturation.

Siblings are recognized as significant influencers of motor skills in infants and young children [28]. Different studies performed on infants have indicated that children with older brothers or sisters show higher motor skill levels [24,27,37], while others have not found significant associations [38,39]. Research conducted with older children is limited, hindering a more in-depth discussion of our findings. Notably, Lopes and Monteiro [40], in a sample of 181 children (mean age 6.1 years old), reported similar results to ours. In their study, sibling characteristics and siblinghood relationships (having older brothers or sisters, birth order) were not significantly associated with MC. In contrast, research on physical fitness levels in children and adolescents has indicated that siblings play a significant role [41,42].

The impact—whether beneficial or detrimental—of an older sibling on motor skill development can be shaped by several variables. For instance, the older sibling’s age plays a role, as highlighted by Hayashida and Nakatsuka [43] in their study on infants, while sibling gender differences may also be influential, as noted by Chiva-Bartoll and Estevan [44]. An additional factor not addressed in the current study is the age gap between siblings, which has been shown to significantly affect sibling dynamics and interactions [45]. Moreover, the intelligence quotient of siblings may also be relevant; older siblings with higher IQs are more inclined to actively support and contribute to their younger siblings’ development [46]. Finally, it should be noted that having siblings may help a child develop social and personal skills probably by increasing family social interaction [47].

In conclusion, judging from the available scientific evidence, while siblings might influence MC development during infancy, this effect appears to be negligible during childhood and adolescence. In this regard, our results also indicated that relative age is not a variable that affects the relationship between siblings and MC. A possible explanation for these findings might rely on the fact that infancy and early childhood are crucial phases for developing motor competence, and it is during these periods when the influence of siblings and RAE must be more noticeable.

The findings of this research should be interpreted within the context of several limitations. Firstly, this study included a convenience sample recruited from schools in the north of Spain. Therefore, potential findings cannot be generalized to other children. Secondly, MC was assessed by means of a single test. Although this test incorporated both product-and process-oriented measures—providing more robust data than using either approach alone—other studies have employed comprehensive motor skill batteries. Such batteries enable a more thorough and nuanced correlational analysis. Thirdly, data on siblings were collected from a subsample, and a smaller percentage of primary school children reported having siblings, which limited the ability to make comparisons with their secondary school peers. Fourthly, the cross-sectional nature of the study limited the ability to establish causal relationships or track changes in motor competence over time. Future research using longitudinal designs is needed to better understand developmental trajectories. Finally, various confounding variables that can influence the development of motor competence, such as sociodemographic and cultural factors or levels of physical activity, were not accounted for in the analysis. Future research should recruit larger, more diverse longitudinal cohorts and employ comprehensive, multi-component motor skill batteries while systematically collecting familial, sociodemographic, and physical activity data to rigorously adjust for confounders; this approach will help clarify developmental trajectories and causal influences on motor competence.

## 5. Conclusions

This study offers a novel perspective by jointly examining relative age and sibling effects on motor competence in both primary and secondary school students in Spain, providing new insights that may inform future educational strategies and research in developmental and school-based contexts.

Preliminary scientific evidence indicates that relative age affects motor competence in primary but not in secondary school children, although its impact is of a low magnitude. Having siblings was not significantly associated with motor competence proficiency in this sample. These results imply that physical education teachers should be aware of the effect of relative age in primary education, not only when assessing motor competence but also when designing inclusive and developmentally appropriate teaching strategies to support younger students within the same academic cohort. In contrast, in secondary education, where the impact of relative age appears to be less significant, this factor may be less critical for both assessment and teaching practice. The present results also imply that curricula and motor skill assessments should be adapted to accommodate developmental variability within the same age cohort. This might include differentiated instruction, flexible grouping, or age-adjusted performance benchmarks to ensure fairness and inclusion. Future longitudinal studies tracking children over time are needed to offer a deeper insight into how relative age might affect fitness in the long term.

## Figures and Tables

**Table 1:** CAMSA scores stratified by age and educational level.

		CAMSA Score
Age (Years)	n (%)	Median (IQR)
6	60 (8.03)	11 (8; 14)
7	51 (6.82)	14 (12; 17)
8	53 (7.10)	16 (14; 19)
9	49 (6.56)	17 (15; 20)
10	76 (10.17)	21 (19; 24)
11	90 (12.05)	23 (19; 25)
12	88 (11.78)	23 (21; 25)
13	127 (17.00)	24 (22; 25)
14	48 (6.43)	25 (22; 26)
15	88 (11.78)	23 (21; 25)
16	17 (2.28)	23 (21; 24)
Educational level		
Primary school	381 (51.00)	18 (14; 22)
Secondary school	366 (49.00)	23 (22; 25)
Total	747	22 (17; 24)

CAMSA scores range from 1 to 28, with higher scores indicating better performance. IQR = interquartile range.

**Table 2:** CAMSA percentile according to birth trimester and educational level.

	CAMSA Score					
	Primary School (*p* = 0.017)		Secondary School (*p* = 0.218)		Total (*p* = 0.007)	
Birth Trimester	n	Median (IQR)	n	Median (IQR)	n	Median (IQR)
1st	78	53 (20; 70)	85	46 (23; 68)	163	46 (20; 69)
2nd	94	54 (34; 78)	93	53 (25; 68)	187	54 (31; 73)
3rd	101	42 (19; 70)	96	37 (16; 66)	197	40 (18; 67)
4th	108	36 (11; 65)	92	46 (26; 67)	200	37 (18; 67)

Comparison of CAMSA percentile among birth trimesters with Kruskal–Wallis test. IQR = interquartile range.

## Data Availability

The raw data supporting the conclusions of this article will be made available by the authors on request, due to privacy considerations.
